# Rational design, synthesis, and antimicrobial evaluation of novel 1,2,3-triazole hybrids: structure–activity relationships, molecular docking, and DFT studies

**DOI:** 10.1038/s41598-026-62695-w

**Published:** 2026-08-11

**Authors:** Mohammed N. Sallam, A. M. A. Hassan, Abdullah Yahya Abdullah Alzahrani, Hossam M. El-Masry, Abeer M. El-Naggar, Eslam M. Abbass

**Affiliations:** 1 Department of Chemistry, Faculty of Science, Ain Shams University, Abbassia, Cairo, 11566 Egypt; 2 Department of Chemistry, Faculty of Science, King Khalid University, 61413 Abha, Saudi Arabia; 3 Chemistry of Natural and Microbial Products Department, Pharmaceutical and Drug Industries Research Institute, Cairo, Egypt

**Keywords:** 1,2,3-Triazole derivatives, Rational drug design, SAR, Docking, DFT, ADME, Antimicrobial activity, Biochemistry, Chemistry, Computational biology and bioinformatics, Drug discovery

## Abstract

A series of novel 2,4-dichlorophenyl–1,2,3-triazole derivatives and their structural hybrids incorporating chalcone, hydrazone, thio/semicarbazide, carbamate, and pyrazole moieties were rationally designed, synthesized, and characterized. The antimicrobial activity of the synthesized compounds was evaluated against Gram-positive and Gram-negative bacteria, as well as *Candida albicans*, revealing clear structure-dependent activity profiles. Among the tested compounds, the parent triazole–acetyl scaffold (compound **3**) exhibited the most consistent broad-spectrum activity, with MIC values of 22.5 mg/mL against *E. coli*, *H. pylori*, and *C. albicans*, and 11.25 mg/mL against *B. cereus* and *S. aureus*. Structure–activity relationship (SAR) and ADME analyses indicated that a balance between lipophilicity and polarity plays a critical role in modulating antimicrobial performance and membrane permeability. Molecular docking studies against *Staphylococcus aureus* topoisomerase IV (PDB: 4URN) revealed favorable binding interactions of the active compounds within the enzyme active site. Complementary density functional theory (DFT) calculations provided insights into electronic properties and reactivity trends, supporting the observed biological behavior. Overall, the results suggest that the 2,4-dichlorophenyl–1,2,3-triazole scaffold represents a promising starting point for further optimization, with antimicrobial activity governed by a balance between electronic properties and physicochemical factors influencing membrane permeability.

**Supplementary Information:**

The online version contains supplementary material available at 10.1038/s41598-026-62695-w.

## Introduction

The rapid emergence of antimicrobial resistance (AMR) has created a critical global health challenge, with multidrug-resistant bacteria and fungi compromising the effectiveness of current therapeutic options. According to recent estimates, drug-resistant infections may cause up to 10 million deaths annually by 2050 if new antimicrobial strategies are not developed^1^. This urgent scenario has driven extensive research into heterocyclic scaffolds, particularly nitrogen-containing systems, which frequently underpin clinically approved antimicrobials^2,3^.

Among these, triazoles represent a privileged structural class with broad pharmacological versatility, such as anticancer^4^, antibacterial^5–7^, antifungal^8,9^, anti-inflammatory^10,11^, anti-tuberculosis^12,13^, anti-convulsion^14^, antimalarial^15,16^, antiviral^17,18^, anti-oxidation^19,20^, and so on. They exist in two isomeric forms, 1,2,3-triazole and 1,2,4-triazole, both characterized by high metabolic stability, strong hydrogen-bonding capacity, and the ability to mimic bioactive functionalities such as amides and carboxylates^21^.

**Fig. 1:**
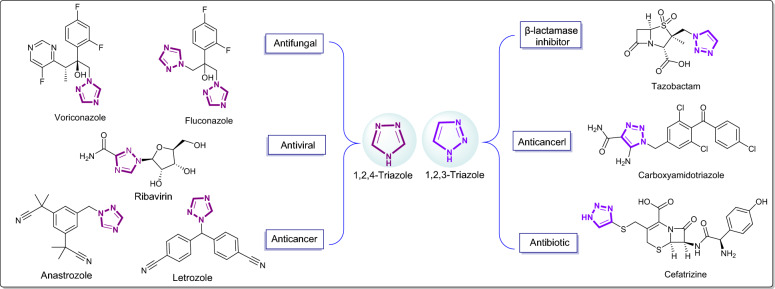
Clinically approved triazole-containing drugs highlighting therapeutic diversity.

Clinically, triazole derivatives occupy a prominent place in modern therapeutics, as illustrated in Fig. 1. The 1,2,4-triazole scaffold is particularly well established, with notable representatives including the antifungal agents fluconazole, itraconazole, and voriconazole, the antiviral ribavirin, the anticancer drugs letrozole and anastrozole, and central nervous system medications such as alprazolam and trazodone^22^. The clinical success of these agents is largely attributable to the triazole ring’s ability to engage critical microbial and enzymatic targets, including DNA gyrase, topoisomerase IV, and sterol 14-α-demethylase^21^.

By comparison, the 1,2,3-triazole isomer is less represented among marketed drugs but has emerged as a scaffold of growing importance. Tazobactam, a widely used β-lactamase inhibitor, incorporates a 1,2,3-triazole moiety that broadens its inhibitory profile when combined with β-lactam antibiotics. Carboxyamidotriazole (CAI), originally developed as an antineoplastic agent, exploits the 1,2,3-triazole ring to interfere with calcium signaling and is under investigation for both anticancer and anti-inflammatory applications. Likewise, Cefatrizine, a semi-synthetic cephalosporin antibiotic, employs a 1,2,3-triazole substituent that enhances antibacterial activity against Gram-positive and Gram-negative strains. Collectively, these examples underscore the therapeutic versatility and untapped potential of 1,2,3-triazoles, positioning them as promising scaffolds for the development of new antimicrobial agents.

### Rational

Several 1,2,3-triazole derivatives have been extensively reported for their antimicrobial potential through diverse mechanisms, with the notable advantage of metabolic stability, low cytotoxicity, and the ability to form strong hydrogen-bonding and π–π interactions with microbial enzymes^21^.

For instance, acridone–1,2,3-triazole hybrids **I** have demonstrated remarkable antibacterial potency against *Staphylococcus aureus*, with MIC values of 27 μM, approaching the activity of fluoroquinolone standards^23^. Similarly, chalcone–1,2,3-triazole hybrids **II** exhibited outstanding activity against *Escherichia coli*, with MIC values as low as 0.003 μM, surpassing ciprofloxacin (MIC: 0.0047 μM) in certain assays^24^.

Furthermore, isatin–1,2,3-triazole conjugates **III** were reported to potently inhibit *S. aureus* with an inhibition zone of 17 mm via binding to the active site of dihydrofolate reductase (DHFR), confirming the role of the triazole moiety as a key pharmacophoric element^25^. A comprehensive review of recent studies reveals that electron-withdrawing substituents (such as nitro or halogen groups) attached to 1,2,3-triazole hybrids generally enhance antibacterial activity, while methoxy substituents frequently improve antifungal potency through favorable interactions with sterol 14-α-demethylase.

**Fig. 2:**
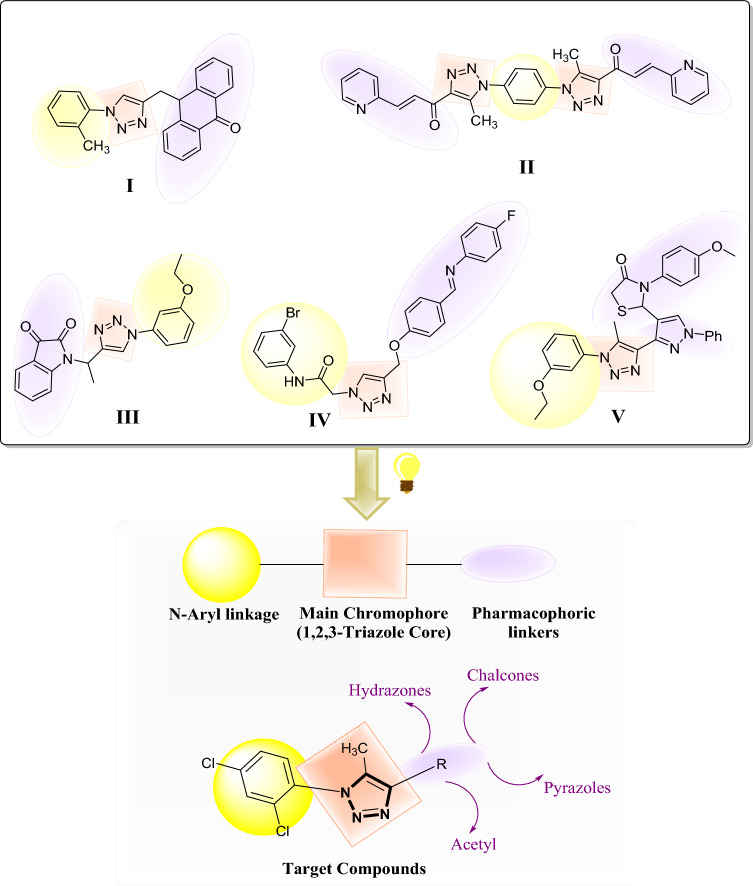
Structural inspiration (**I**–**V**) and rational design of new 1,2,3-triazole-based antimicrobial hybrids.

The utility of the 1,2,3-triazole scaffold is further emphasized by its successful application in the design of multifunctional hybrids. For example, Azomethine–triazole conjugates have shown enhanced binding affinity through imine linkages **IV,** which have an MIC of 12.3 μM against the gram-positive bacterium *B. subtilis*^26^. In contrast, pyrazole–triazole derivatives are known to reinforce antimicrobial activity through additional heteroaryl interactions, showing significant activity equal to that of the standard drug ampicillin with an MIC of 2.60 μg/mL **V**^27^. These findings underscore the versatility and pharmacological significance of the 1,2,3-triazole core as a building block for novel antimicrobial agents, as illustrated in Fig. 2.

Guided by this evidence, the present study describes the design, synthesis, and characterization of novel 1,2,3-triazole derivatives and their structural hybrids, including chalcones, hydrazones (Azomethine), and pyrazoles. Their antimicrobial potential was assessed against representative Gram-positive, Gram-negative, and fungal pathogens, complemented by molecular docking studies on *S. aureus topoisomerase* IV (PDB: 4URN) and DFT calculations to rationalize electronic and binding properties. This integrated experimental–computational approach aims to identify promising lead candidates and deepen understanding of the SAR governing triazole-based antimicrobial activity.

## Result and discussion

### Chemistry

The target compound 1-(1-(2,4-dichlorophenyl)-5-methyl-1H-1,2,3-triazol-4-yl)ethan-1-one (**3**) was synthesized following previously reported procedures^28^. The synthesis began with the diazotization of *2,4-dichloroaniline* (**1**) using sodium nitrite and hydrochloric acid, followed by the addition of sodium azide to yield *1-azido-2,4-dichlorobenzene* (**2**). Subsequent reaction of the azide intermediate **2** with acetylacetone afforded the target acetyl triazole derivative **3**, which served as a key intermediate for further transformations. Structurally, compound **3** was confirmed by IR through the characteristic acetyl-group absorption at 1686 cm⁻^1^. The ^1^H-NMR spectrum further supports this assignment, showing two distinct methyl signals: a CH₃ resonance at 2.37 ppm and a COCH₃ resonance at 2.56 ppm.

**Scheme 1:**
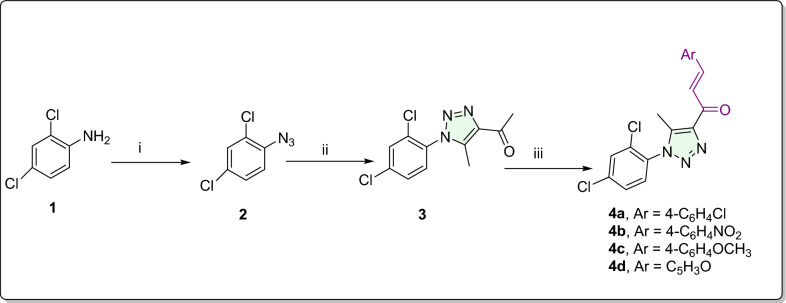
Synthetic procedure of compounds **2-(4a-d)** Reagents/conditions: (i) HCl–NaNO_2_, NaN_3_/ ice bath; (ii) acetylacetone, K_2_CO_3_, 75 °C, 4 h (Yield = 93%); (iii) Ar-CHO, NaOH (10%), EtOH, rt, 4 h (Yield = 48–77%).

The bifunctional nature of compound **3**, possessing both an electrophilic carbonyl group (C = O) and a nucleophilic active methyl group (–COCH₃), renders it reactive toward both nucleophilic and electrophilic reagents, facilitating the synthesis of various triazole derivatives. To explore its nucleophilic reactivity and to investigate the influence of electronic factors on subsequent biological activity, compound **3** was reacted with a series of aromatic and heteroaromatic aldehydes bearing electron-donating groups (e.g., –OCH₃), electron-withdrawing groups with mesomeric effects (e.g., –NO₂), inductively withdrawing groups (e.g., Cl), as well as heterocyclic aldehydes such as furfural. These reactions were conducted in the presence of sodium hydroxide, which enhances the nucleophilicity of the methyl group. The corresponding chalcone derivatives (**4a–d**) were obtained in each case Scheme 1.

The structures of the synthesized triazole-based chalcone derivatives (**4a–d**) were confirmed by spectral analysis. The IR spectra displayed characteristic absorption bands for the chalcone carbonyl group (C = O) in the range of 1653–1664 cm⁻^1^, consistent with conjugated ketone functionality. In the ^1^H NMR spectra, signals corresponding to aromatic protons were observed in the region of 7.02–8.93 ppm. Additionally, the methyl group attached to the triazole ring appeared as a singlet in the range of 2.45–2.51 ppm. In compound **4c**, the methoxy group was identified as a distinct singlet at 3.83 ppm, further supporting the proposed structure.

**Scheme 2:**
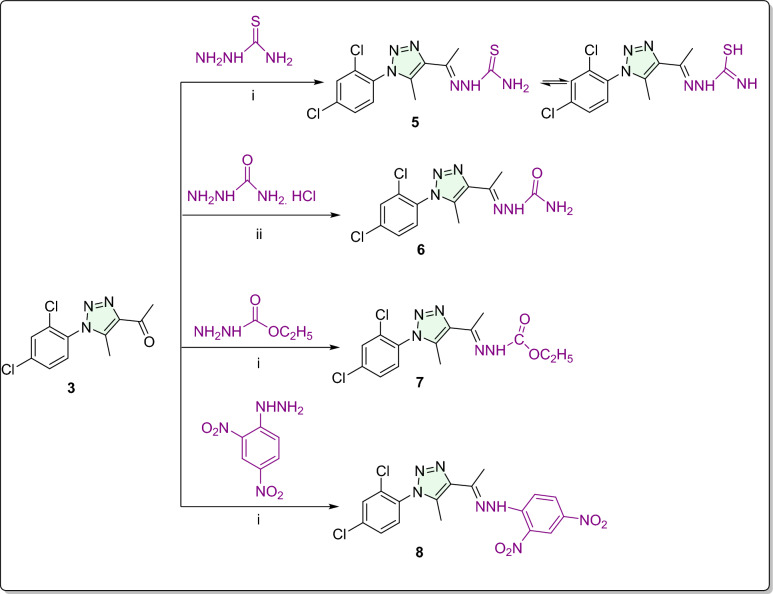
Synthetic procedure of compounds **5–8** Reagents/conditions: (i) Absolute ethanol, glacial acetic acid, reflux, 4 h,(ii) Absolute ethanol, Sodium acetate, reflux, 4 h, yield: 64–79%.

Furthermore, to investigate the electrophilic behavior of the acetyl triazole derivative (**3**), it was reacted with various hydrazide derivatives in the presence of acetic acid. The acidic medium serves to activate the carbonyl group of the acetyl moiety by protonating the carbonyl oxygen, thereby increasing the positive character on the carbon atom and enhancing its susceptibility to nucleophilic attack. This activation facilitates the condensation reaction with nucleophilic hydrazides, leading to the formation of the corresponding hydrazone derivatives **5–8 **Scheme2.

The incorporation of thioamide (as in compound **5**) and amide linkages (as in compounds **6** and **7**) was strategically designed to evaluate their potential binding affinity against microbial targets. These structural modifications were introduced to assess how variations in the linker functionality influence antimicrobial activity, and to enable a comparative analysis with compound **8**, which contains only a simple amine (–NH) linkage. Structurally, the synthesized compounds were confirmed through spectral analysis.

The IR spectra supported the successful transformations by showing the disappearance of the carbonyl (C = O) band of the acetyl group and the appearance of characteristic bands corresponding to new functional groups, such as hydrazone and amide linkages. For example, compound **5** exhibited stretching bands at 3284 and 3211 cm^–1^, corresponding to NH and NH₂ groups, respectively. Compound **6** showed absorption bands at 3293, 3186, and 3145 cm^–1^, consistent with NH₂ and NH groups, along with a distinct amide carbonyl (C = O) band at 1673 cm⁻^1^. Similarly, compound **7** displayed a band at 3103 cm^–1^ attributed to the NH group and a strong absorption at 1708 cm^–1^ corresponding to the ester carbonyl (COOEt) group, confirming the successful formation of the desired structures.

The ^1^H NMR spectra of the synthesized hydrazone derivatives confirmed the presence of NH and NH₂ protons as labile signals, which were exchangeable upon addition of D₂O, indicating their involvement in hydrogen bonding and proton exchange. In compound **7**, the ethyl group of the ester moiety appeared as a characteristic triplet at 1.24 ppm, corresponding to the terminal –CH₃ group, and a quartet at 4.13 ppm, corresponding to the adjacent –CH₂– group, confirming the presence of the ethyl ester functionality (–CH₂CH₃). Additionally, in compound **8**, the aromatic protons of the dinitrobenzene ring were observed in the downfield region, appearing as two doublets at 8.12, 8.19 ppm, and a singlet signal at 8.93 ppm, consistent with the deshielding effect of the electron-withdrawing nitro groups.

**Scheme 3:**
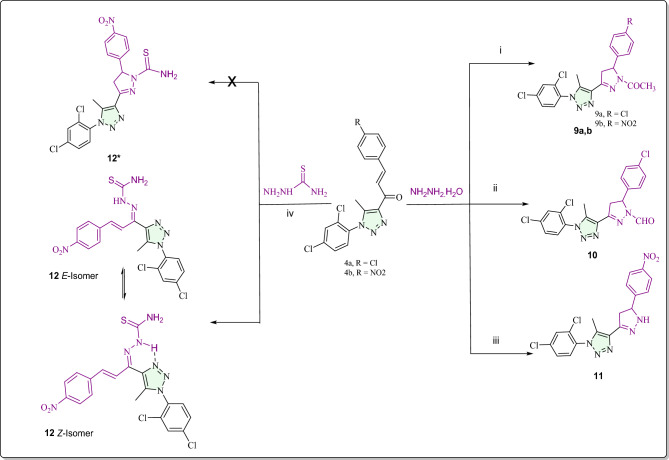
Synthetic procedure of compounds **9a,b-12** Reagents/conditions: (i) Absolute ethanol, glacial acetic acid, reflux, 6 h, yield 65% **9a**, 75% **9b** (ii) Absolute ethanol, Formic acid, reflux, 6 h, yield 64% (iii) Absolute ethanol, reflux, 6 h, yield 60% (iv) Absolute ethanol, glacial acetic, reflux, 6h, yield 65%.

Due to the well-known reactivity of chalcones with hydrazine hydrate and its derivatives, and considering the significant biological relevance of the pyrazole ring, the high-yielding chalcone derivatives **4a** and **4b** were selected for further transformation. These compounds were reacted with hydrazine hydrate under different conditions to afford a series of pyrazole derivatives. Treatment with hydrazine hydrate alone led to the formation of unsubstituted pyrazole rings, while reactions carried out in the presence of formic acid and acetic acid yielded the corresponding N-formyl pyrazole and N-acetyl pyrazole derivatives, respectively Scheme 3. All synthesized pyrazole derivatives **9–11** were confirmed by spectral data. In the H NMR spectra, the two hydrogens of the (CH–CH₂) moiety of the pyrazole ring appeared as doublets of doublets in the range δ 2.99–4.12 ppm, while the corresponding CH proton resonated as a doublet of doublets at δ 4.99–5.57 ppm.

For compounds **9a,b**, the presence of the N-acetyl group was supported by characteristic IR stretching bands at 1657 and 1666 cm⁻^1^, respectively, and by singlet signals at δ 2.46 ppm in the H NMR spectra.

The N-formyl group in compound **10** was confirmed by an IR absorption band at 1676 cm⁻^1^ and a singlet signal at δ 8.90 ppm in the H NMR spectrum, corresponding to the N–CHO proton.

In contrast, the reaction of chalcone **4b** with thiosemicarbazide did not yield the expected pyrazole derivative 12*; instead, it produced a mixture of *E/Z* isomers of the corresponding thiosemicarbazone derivative **12**. The presence of *E/Z* isomerism was clearly evidenced by the H NMR spectrum, which shows duplication of signals arising from the same protons experiencing different magnetic environments in the two geometric isomers. The methyl group appeared as two distinct singlet signals at δ 2.16 and 2.26 ppm, corresponding to the *E* and *Z* isomers, respectively, reflecting the sensitivity of the CH₃ group to changes in spatial orientation around the C = N bond. The aromatic protons were observed as overlapping multiplet signals in the range δ 6.96–8.65 ppm, resulting from the superposition of resonances from both isomeric forms.

Most notably, the hydrazinic NH proton displayed two well-resolved singlet signals at δ 10.71 and 11.42 ppm, which provides strong evidence for *E*/*Z* isomerism. The downfield-shifted NH signal is assigned to the *Z*-isomer, which is stabilized by intramolecular hydrogen bonding between the hydrazinic NH proton and the triazole nitrogen. This interaction leads to enhanced deshielding of the NH proton, whereas the upfield signal corresponds to the *E*-isomer, in which such hydrogen bonding is geometrically unfavorable.

### Biological evaluation

#### Antimicrobial and antifungal activity

The antimicrobial evaluation of the newly synthesized 1,2,3-triazole derivatives revealed diverse inhibitory activities against Gram-negative and Gram-positive bacteria as well as *Candida albicans*. These findings underscore the potential of the 2,4-dichlorophenyl–1,2,3-triazole scaffold as a versatile platform, wherein structural modifications at the chalcone, pyrazole, thiosemicarbazide, and carbamate termini critically influenced potency and spectrum.

Broad-Spectrum Activity, Among the tested compounds, **3** emerged as the most consistently active derivative. It inhibited all organisms examined, producing inhibition zones of *E. coli* (14 mm), *H. pylori* (13 mm), *B. cereus* (15 mm), *S. aureus* (13 mm), and *C. albicans* (15 mm). Although its MIC values (11.25–22.5 × 10^3^ µg/mL) were higher than the reference gentamicin (60–125 µg/mL), **3** remains noteworthy for its broad-spectrum efficacy spanning Gram-negative, Gram-positive, and fungal targets.

Gram-Negative Inhibition, Several compounds exhibited selective potency against Gram-negative bacteria. The thiosemicarbazide derivatives **5** and **12** consistently inhibited *E. coli* (12 mm) and *H. pylori* (10–12 mm). Lipophilicity played a decisive role in their spectrum of activity: **5**, with moderate lipophilicity, extended its activity to Gram-positive organisms (*B. cereus* 10 mm, *S. aureus* 14 mm), whereas the bulkier and more lipophilic **12** was restricted to Gram-negative inhibition. This observation aligns with the permeability barrier of Gram-positive bacteria, where excessive lipophilicity can hinder diffusion across the thick peptidoglycan matrix.

**Fig. 3:**
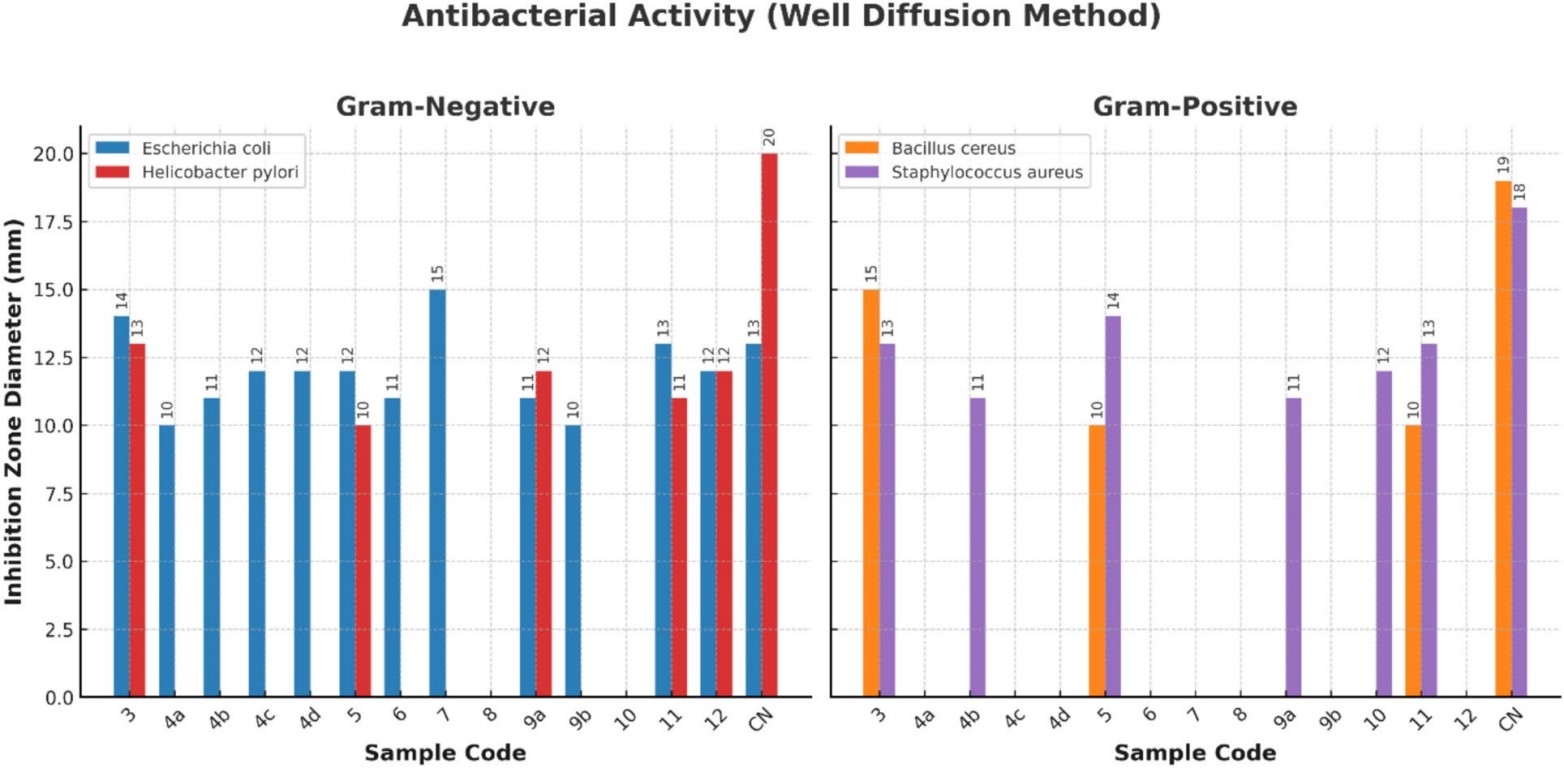
Antibacterial activity of the synthesized compounds determined by the well diffusion method, expressed as inhibition zone diameters (mm) against Gram-negative (*Escherichia coli*, *Helicobacter pylori*) and Gram-positive (*Staphylococcus aureus*, *Bacillus cereus*) bacteria.

Gram-Positive Inhibition, Significant inhibition of *S. aureus* and *B. cereus* was observed with **11** (13 and 10 mm, respectively) and **5**, demonstrating that judicious substituent choice can broaden activity beyond Gram-negatives. Notably, the chalcone derivative **4b** (*p*-NO₂) inhibited *S. aureus* (11 mm), consistent with the established role of electron-withdrawing substituents in enhancing the reactivity of Michael acceptor systems and thereby extending coverage to Gram-positive pathogens, as illustrated in Fig. 3.

**Fig. 4:**
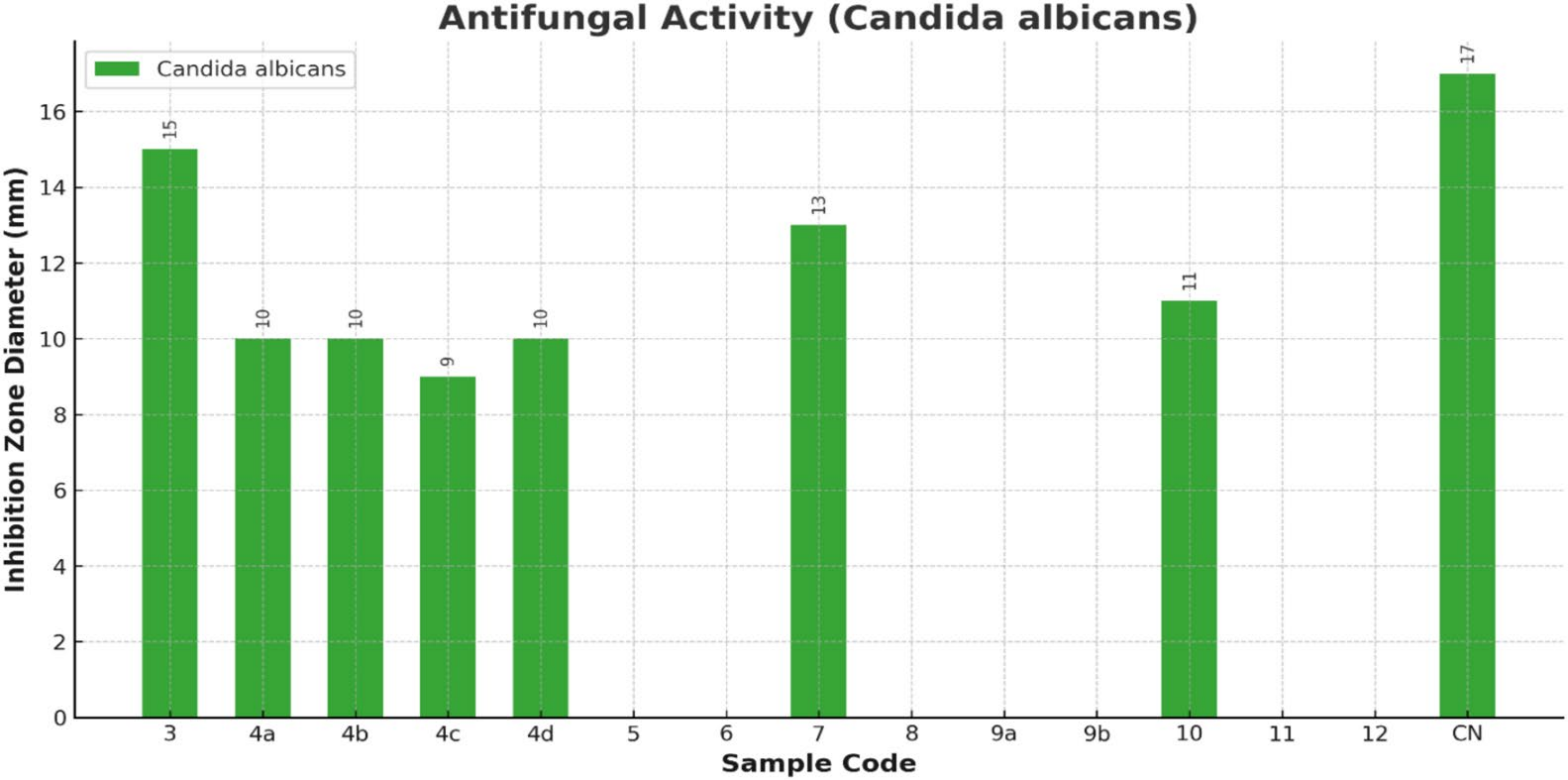
Antifungal activity of the synthesized compounds against *Candida albicans*, determined by the well diffusion method and expressed as inhibition zone diameters (mm).

Antifungal Activity, Antifungal evaluation against *C. albicans* revealed potent effects for **3** (15 mm), **7** (13 mm), and **10** (11 mm). Among these, **7** (a carbamate hydrazone) was particularly significant, as it combined antibacterial activity (*E. coli* 15 mm) with pronounced antifungal effects, suggesting that increased polarity and solubility associated with carbamate substitution may facilitate fungal penetration. Interestingly, **10** (an N-formyl pyrazole) exhibited exclusive antifungal activity, underscoring how subtle modifications at the pyrazole nitrogen can redirect selectivity toward fungal targets. Chalcone derivatives also showed modest antifungal effects (9–10 mm), indicating that the conjugated chalcone system provides a baseline level of fungal inhibition that can be optimized through substituent tuning, as illustrated in Fig. 4.

Comparison with Reference Drug, Gentamicin, employed as the positive control, displayed the largest inhibition zones overall (*H. pylori* 20 mm, *B. cereus* 19 mm, *S. aureus* 18 mm, *E. coli* 13 mm, *C. albicans* 17 mm) and significantly lower MIC values compared to the synthesized compounds, as illustrated in Table 1. Nevertheless, while gentamicin served as a benchmark for potency, the triazole derivatives offered valuable insights into scaffold-specific tuning, particularly with respect to fungal inhibition, an activity absent in the reference antibiotic. Although compound 3 demonstrated consistent broad-spectrum activity, its MIC values remain significantly higher than those of clinically used antibiotics, indicating relatively low potency. Therefore, these compounds should be regarded as preliminary scaffolds rather than optimized antimicrobial agents. This reduced activity is likely associated with physicochemical limitations such as suboptimal membrane permeability and polarity, as supported by the ADME analysis. Future work will focus on structural optimization to enhance potency, including modification of substituents, tuning of lipophilicity–polarity balance, and further structure–activity relationship studies. In addition, formulation strategies or prodrug approaches may be explored to improve biological performance.

**Table 1:** The minimal inhibitory concentration (MIC) of the tested sample applying the Micro dilution broth method.

Test bacteria	Minimal inhibitory concentration (MIC)	
	Sample concentration	
	(µg/mL)	(µg/mL)
	3	Gentamicin (CN)
***Escherichia coli***	22.5 × 10^3^	94.0
***Helicobacter pylori***	22.5 × 10^3^	125.0
***Bacillus cereus***	11.25 × 10^3^	125.0
***Staphylococcus aureus***	11.25 × 10^3^	118.0
***Candida albicans***	22.5 × 10^3^	60.0

##### SAR study

All synthesized compounds shared the 2,4-dichlorophenyl–1,2,3-triazole pharmacophore, which provided a consistent antibacterial baseline. Modifications at the terminal moieties chalcones, pyrazoles, thiosemicarbazides, semicarbazides, and carbamates significantly influenced potency and selectivity.

The chalcone derivatives **4a-d** were primarily active against *E. coli* (10–12 mm) and showed modest antifungal effects (9–10 mm). The p-nitro chalcone **4b** broadened activity to *S. aureus* (11 mm), highlighting the role of electron-withdrawing groups in enhancing the electrophilicity of the α,β-unsaturated carbonyl and extending the antibacterial spectrum. In contrast, OMe and furyl substituents improved Gram-negative activity but not antifungal potency.

The pyrazole derivatives demonstrated marked sensitivity to N-substitution. The unsubstituted analogue **11** exhibited broad-spectrum inhibition (*E. coli*, *H. pylori*, *B. cereus*, *S. aureus*). N-acetylation produced divergent outcomes: **9a** (*p*-Cl) retained balanced activity, while **9b** (*p*-NO_2_) lost Gram-positive coverage. Notably, N-formylation **10** redirected activity exclusively to *Candida albicans* (11 mm), confirming that small modifications at the pyrazole nitrogen dramatically alter selectivity.

The thiosemicarbazide derivatives **5** and **12** consistently inhibited Gram-negative bacteria. **5**, with moderate lipophilicity, extended its spectrum to Gram-positive strains (*B. cereus*, *S. aureus*), whereas the bulkier, more lipophilic **12** was restricted to Gram-negative activity. This underscores the need to balance lipophilicity to achieve broader antimicrobial coverage.

Finally, the semicarbazide and carbamate analogues revealed the impact of carbonyl substitution. The semicarbazide **6** showed only modest *E. coli* inhibition, while the carbamate **7** exhibited dual antibacterial (*E. coli* 15 mm) and antifungal (*C. albicans* 13 mm) activity. Enhanced polarity and solubility from the carbamate moiety likely improved fungal penetration without compromising antibacterial potency.

**Fig. 5:**
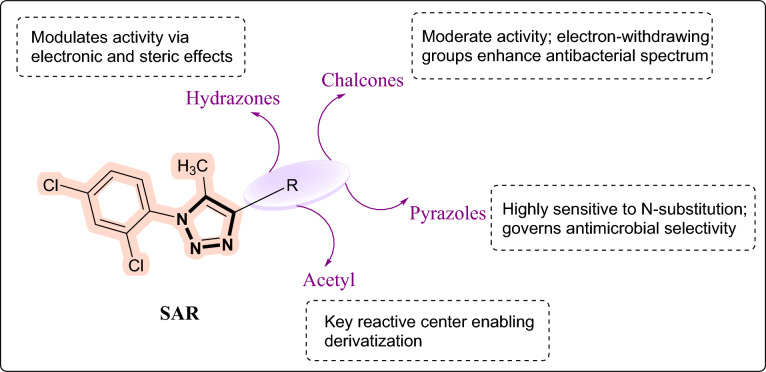
Schematic illustration of the structure–activity relationships (SAR) of the synthesized 1,2,3-triazole derivatives.

Collectively, these findings demonstrate that the 2,4-dichlorophenyl–1,2,3-triazole scaffold represents a versatile antibacterial platform, as illustrated in Fig. 5. Fine-tuning electronic properties (NO_2_ vs OMe/Cl), lipophilicity, and N-substitution provides a rational framework for developing triazole-based derivatives with tailored activity profiles against Gram-negative, Gram-positive, or fungal pathogens.

### In silico studies

#### Molecular docking

Molecular docking was employed to rationalize the antimicrobial activity of the synthesized triazole derivatives (**3**–**12**) against *S. aureus* topoisomerase IV (PDB ID: 4URN). This enzyme was selected because it is a validated antibacterial target co-crystallized with novobiocin, an established inhibitor. Topoisomerase IV is essential for bacterial DNA replication, and 4URN has been widely applied in docking studies of triazole derivatives, making it a reliable model for structure–based evaluation. The docking results are therefore interpreted qualitatively to suggest possible binding modes rather than definitive target specificity.

Docking scores ranged from − 6.19 to − 6.90 kcal/mol compared with novobiocin (− 7.18 kcal/mol), as illustrated in Table 2. The N-acetyl pyrazoles (**9a**, **9b**) exhibited the best affinities (− 6.90 and − 6.84 kcal/mol), while the N-formyl pyrazole (**10**) and pyrazole derivative (**11**) showed moderate binding (− 6.69 and − 6.59 kcal/mol). Among the hydrazone derivatives (**5**–**8**), compound **6** displayed relatively good binding (− 6.70 kcal/mol), whereas compound **8** was the weakest binder. The chalcone derivatives (**4a**–**4d**) and precursor (**5**) gave the lowest scores (− 6.19 to − 6.31 kcal/mol). It is important to note that the differences in docking scores among the tested compounds are relatively small (within ~ 0.7 kcal·mol⁻^1^), which falls within the typical uncertainty range of AutoDock Vina scoring functions. Therefore, these values should be interpreted qualitatively rather than quantitatively. Accordingly, the docking results are primarily used to suggest possible binding modes and key ligand–protein interactions rather than to establish definitive activity rankings.

**Table 2:** Molecular docking scores (kcal/mol) and RMSD values of compounds **3**–**12** against *S. aureus* topoisomerase IV (PDB: 4URN), compared with the co-crystallized ligand.

Compound	Docking score	RMSD	Compound	Docking score	RMSD
**3**	− 6.49	1.38	***7***	− 6.62	1.51
**4a**	− 6.27	1.37	***8***	− 6.57	0.86
**4b**	− 6.25	2.05	***9a***	− 6.90	1.33
**4c**	− 6.31	1.57	***9b***	− 6.84	1.22
**4d**	− 6.30	1.63	***10***	− 6.69	1.50
**5**	− 6.65	1.85	***11***	− 6.59	1.95
**6**	− 6.70	0.89	***12***	− 6.37	1.89
**Co-Crystal**	− 7.18	1.31	**Gentamicin**	− 6.17	1.76

**Fig. 6:**
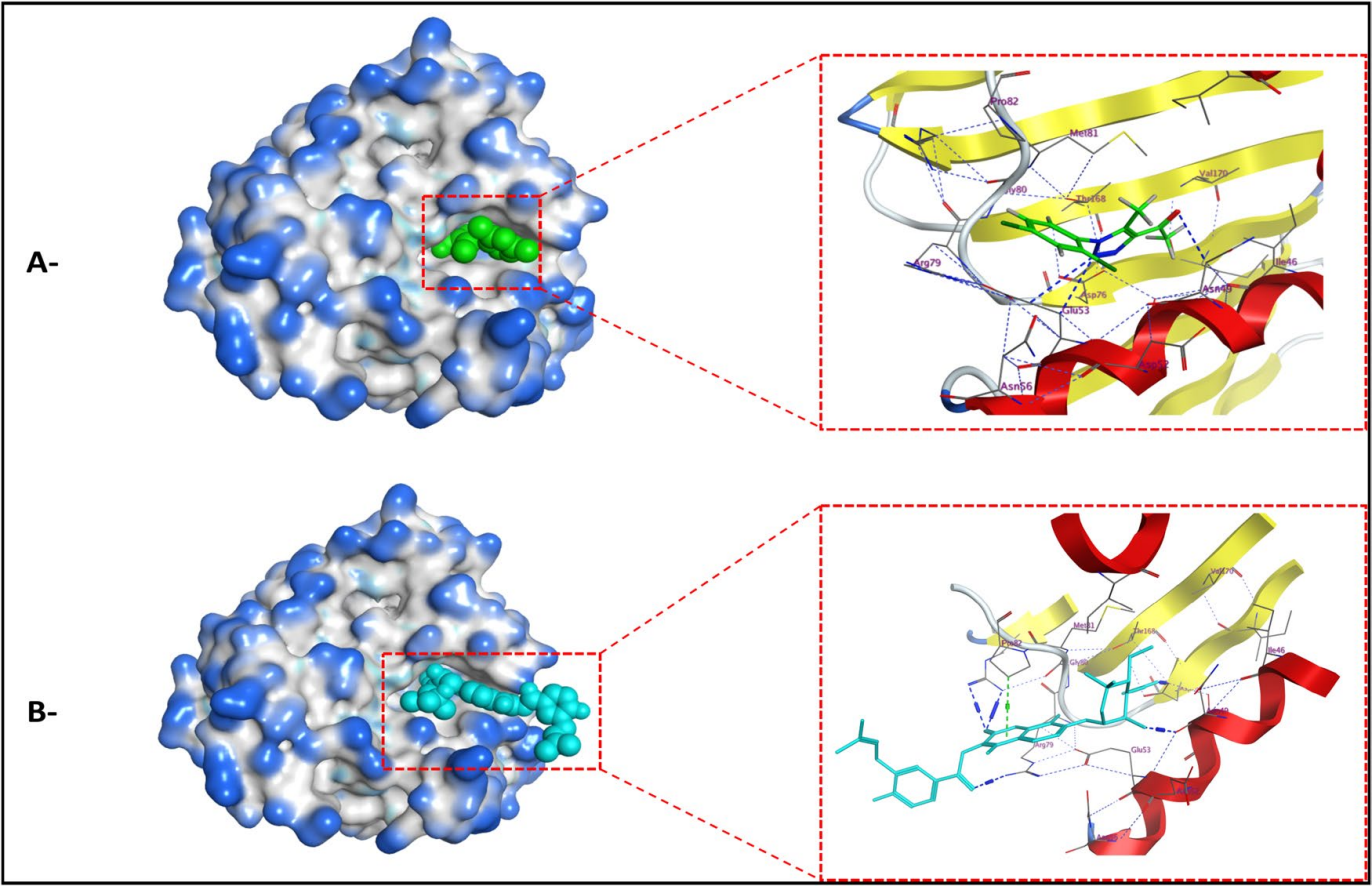
Binding poses of compound **3** (A) and the co-crystal ligand (B) in the active site of *S. aureus* topoisomerase IV (PDB: 4URN).

Importantly, the triazole–acetyl core (compound **3**), which demonstrated the broadest antimicrobial activity in vitro, was confirmed to occupy the same pocket as novobiocin, forming H-bonds with Asn49 and Glu53. In contrast, novobiocin engages multiple residues (Asn49, Asp76, Arg79, Arg138) and establishes π–π stacking with Pro82, explaining its slightly stronger binding affinity. Overlay with the co-crystal validated the conserved orientation of compound **3** inside the catalytic cavity, as illustrated in Fig. 6.

**Fig. 7:**
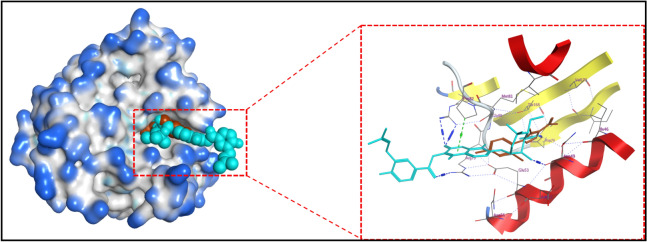
Binding-site superposition of compound **3** (brown) and the co-crystalized ligand (cyan) in *S. aureus* topoisomerase IV (PDB: 4URN).

**Fig. 8:**
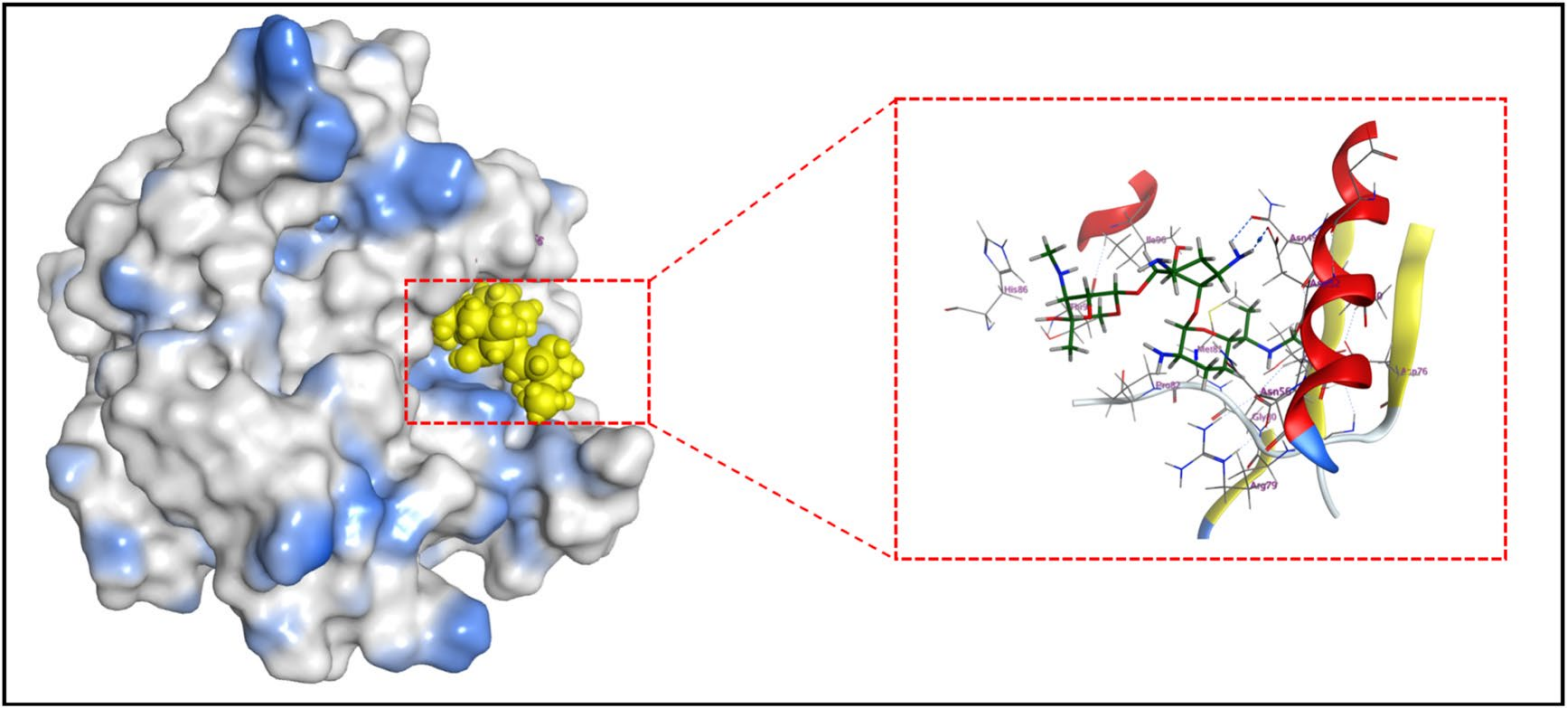
Binding poses of Gentamicin in the active site of *S. aureus* topoisomerase IV (PDB: 4URN).

The triazole–acetyl core (compound **3**) was confirmed to occupy the same binding pocket as the co-crystallized ligand novobiocin, reproducing the key Asn49 anchoring interaction, as illustrated in Fig. 7. Modifications around this scaffold significantly influenced both docking and antimicrobial profiles. Increasing polarity, as in the hydrazone derivatives (**5**–**8**) and the N-acetyl pyrazoles (**9a**, **9b**), improved binding affinity in silico, likely due to their additional heteroatoms and hydrogen-bonding capacity. However, this higher polarity may limit membrane permeability, explaining their weaker biological performance compared with compound **3**. Conversely, reducing polarity, as in the chalcone derivatives (**4a**–**4d**), resulted in lower docking scores (− 6.19 to − 6.31 kcal/mol), consistent with their weaker target engagement. Although gentamicin possesses extensive polarity and multiple hydrogen-bonding groups, its relatively large molecular size and highly polar surface appear to hinder complete accommodation within the topoisomerase IV binding pocket. Consequently, a substantial portion of the molecule remains exposed outside the active site, reducing optimal ligand–protein contacts and explaining its comparatively weak docking score despite its strong experimental antimicrobial activity. This trend indicates that fine-tuning polarity is critical to balance target affinity with cellular uptake in future optimization (Fig. 8).

#### DFT calculations

To enable a chemotype-level comparison on a common physicochemical basis, the compound set was stratified into four principal classes: the triazole–acetyl main core, chalcone derivatives, hydrazone derivatives, and pyrazole derivatives. One representative per class was selected for conceptual DFT analysis: the triazole–acetyl main core **3**, chalcone **4b**, **5** hydrazone/thiosemicarbazone, and pyrazole derivative **11**. Frontier orbital energies (E_HOMO/E_LUMO) were calculated and used to derive the standard global reactivity descriptors ionization potential (I), electron affinity (A), electronegativity (χ), chemical potential (μ), global hardness (η), softness (S), and electrophilicity (ω), which were then interpreted comparatively within a single computational protocol, as illustrated in Table 3.

**Table 3:** Calculated DFT frontier molecular orbitals and global chemical reactivity parameters.

Compound	EHOMO (eV)	ELUMO (eV)	GAP (eV)	IP (I) (eV)	EA(A) (eV)	Χ (eV)	μ (eV)	η (eV)	S (eV^-1^)	ω (eV)	Dipole (D)
**3**	− 6.99	− 1.87	5.12	6.990	1.870	4.430	− 4.430	2.560	0.195	3.833	7.14
**4b**	− 7.19	− 3.31	3.88	7.190	3.310	5.250	− 5.250	1.940	0.258	7.104	7.9535
**5**	− 6.02	− 2.04	3.98	6.020	2.040	4.030	− 4.030	1.990	0.251	4.081	6.32
**11**	− 6.54	− 3.09	3.45	6.540	3.090	4.815	− 4.815	1.725	0.290	6.720	6.45

**Fig. 9:**
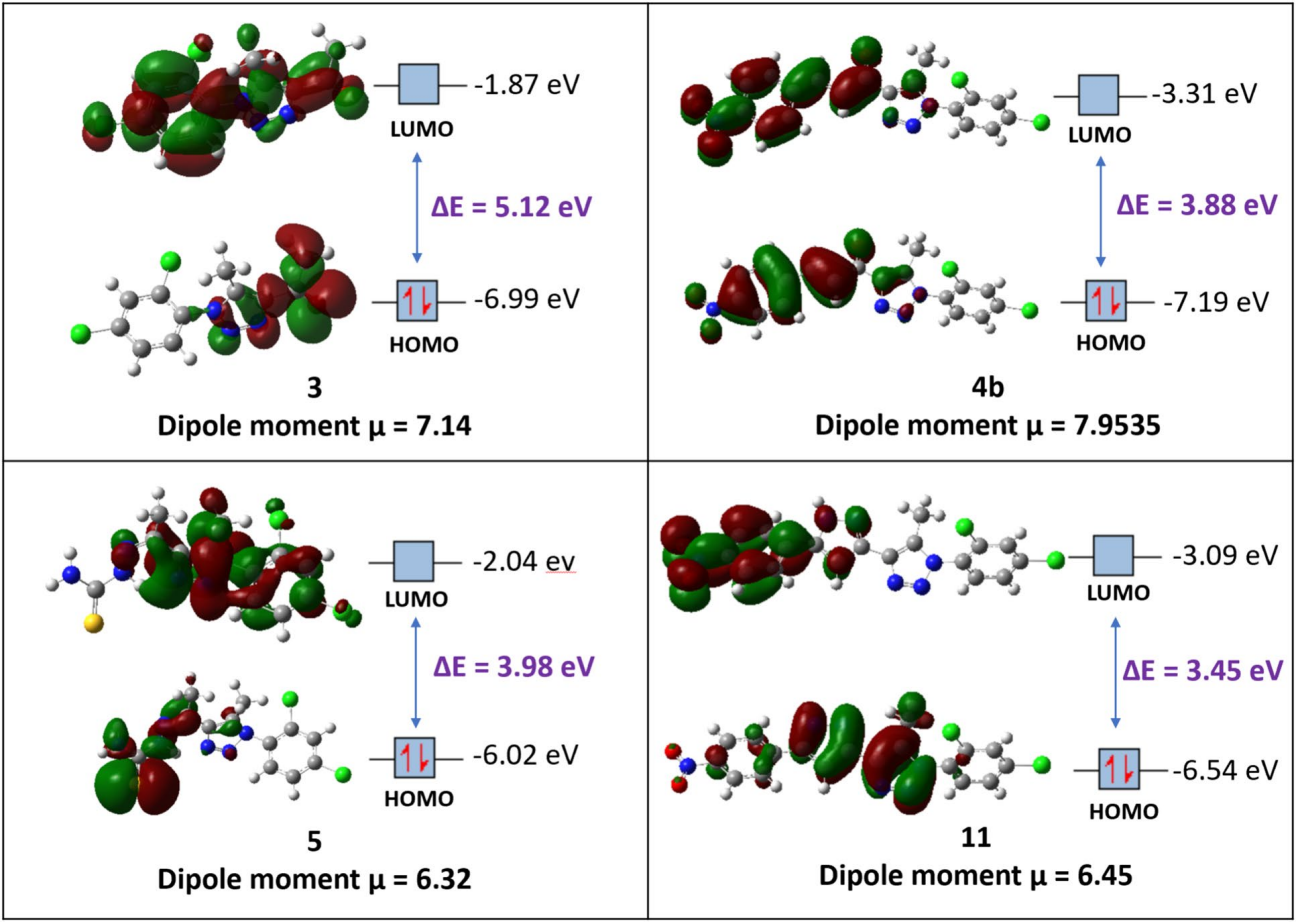
Frontier molecular orbitals (HOMO and LUMO), energy gaps (ΔE), and dipole moments of compounds **3**, **4b**, **5**, and **11** calculated at the B3LYP/6-311+G(d,p) level of theory.

Electronic stability and intrinsic reactivity. The four chemotypes separate clearly by HOMO–LUMO gap and hardness/softness, as illustrated in Fig. 9. **3** displays the largest gap and highest hardness (η), indicating the most electronically “hard,” least polarizable framework. **11** and **4b** show smaller gaps and higher softness (S), implying greater polarizability and a stronger propensity for charge redistribution. **5** sits intermediate, consistent with its additional donor/acceptor functionality. In short, the reactivity/softness trend is **11** ≳ **4b** ≈ **5** < **3**, which provides a convenient electronic hierarchy across scaffolds.

Charge-transfer tendencies and electrophilicity. Consolidated indices that capture electron-acceptor ability (χ, ω) further differentiate the series. **4b** exhibits the highest electronegativity and electrophilicity (ω), followed closely by **11**; **5** is moderate, and **3** is the least electrophilic. This implies that chalcone and pyrazole representatives are more chemically poised to engage in polar interactions (e.g., H-bonding, π-stacking/π–cation) within protein pockets. In contrast, the core is electronically robust and less prone to charge acceptance.

Alignment with docking. These electronic trends are qualitatively consistent with the docking outcomes. The softer, more electrophilic chemotypes (**11**, **5**, **4b**) tend to form richer polar contact networks in the binding site, whereas the harder core 3 scores mid-pack but still achieves credible poses. The chalcone’s slightly weaker docking relative to pyrazole/hydrazone, despite high ω, is readily rationalized by its lower intrinsic H-bonding capacity and heavier reliance on π-acceptor character; the pyrazole and hydrazone, carrying more heteroatoms, better balance polarity and directional interactions.

The chemotype-level electronic hierarchy (softness/electrophilicity: **11** ≳ **4b** **≈ 5 < 3**) rationalizes the docking trend; softer, more electrophilic scaffolds (pyrazole/hydrazone) form richer polar contacts. While the harder, less polar core (**3**) achieves broader whole-cell activity by striking a better balance between permeation and engagement. Thus, DFT explains *why* certain scaffolds dock better yet underperform in cells (polarity/desolvation penalties) and *why* mid-polarity scaffolds translate more consistently. Practically, these insights direct next-round design toward maintaining interaction-enabling softness while tempering dipole/polarity to preserve permeability, turning electronic advantages into cellular potency.

The calculated DFT descriptors, including HOMO–LUMO energy gap, chemical hardness/softness, and electrophilicity index, provide insight into the electronic properties and reactivity of the studied compounds. These parameters may influence the ability of the molecules to interact with biological targets, such as through charge transfer or stabilization of ligand–protein complexes. However, it is important to note that these descriptors do not directly predict antimicrobial activity, as whole-cell activity is governed by additional factors including membrane permeability, solubility, and metabolic stability. Therefore, the DFT results are interpreted qualitatively to support the observed structure–activity relationships rather than as definitive predictors of biological performance.

#### Molecular electrostatic potential (MEP) analysis

**Fig. 10:**
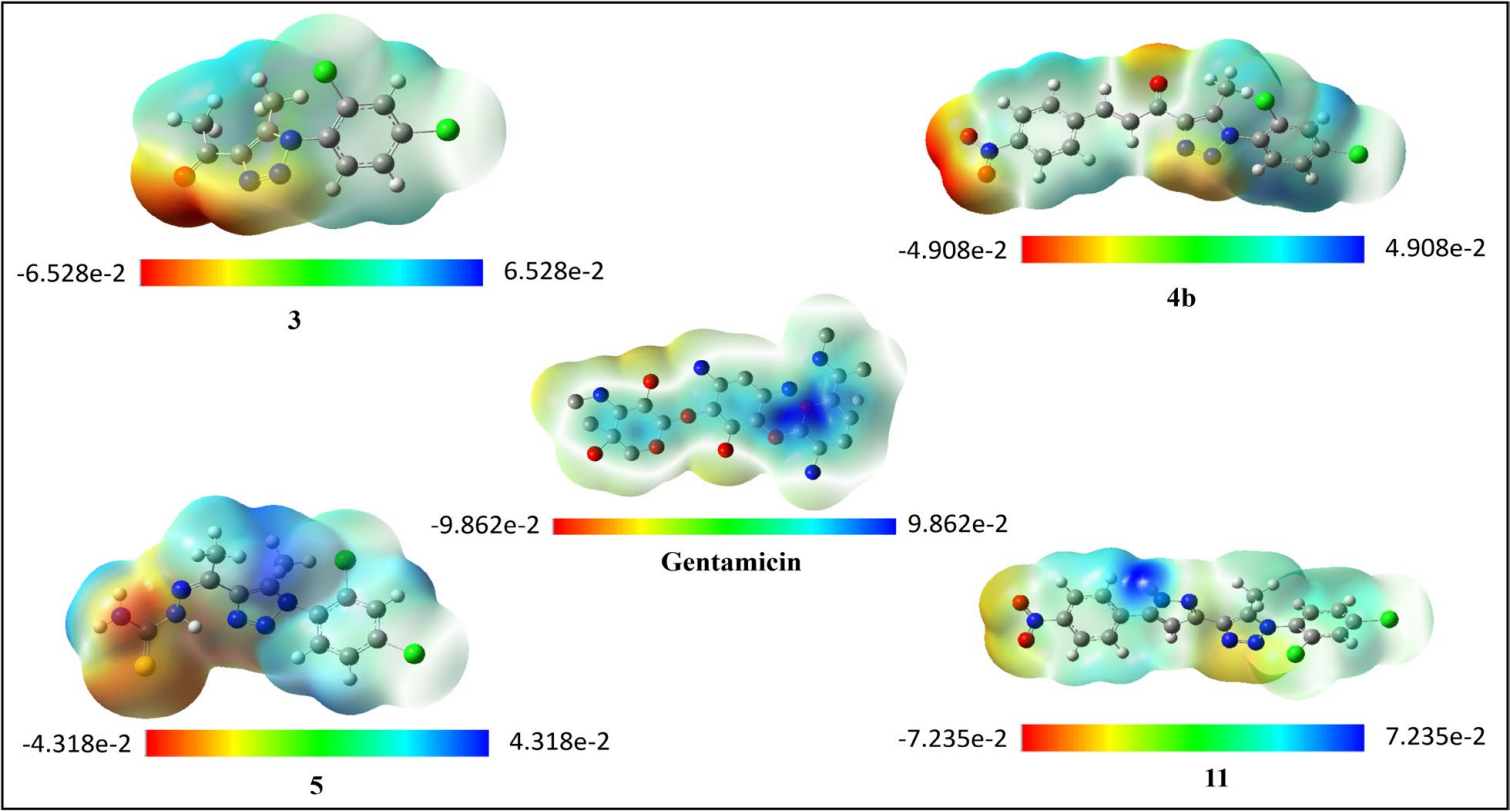
Molecular electrostatic potential (MEP) maps for compounds **3**, **4b**, **5**, **11** and **Gentamicin** plotted on total electron density (isovalue = 0.0004 a.u.).

The Molecular Electrostatic Potential (MEP) maps generated for the four representative chemotypes, core triazole–acetyl scaffold (**3**), chalcone (**4b**), hydrazone/thiosemicarbazone (**5**), and pyrazole derivative (**11**), offer a comparative assessment of surface charge distribution across these structural frameworks, as illustrated in Fig. 10. In these maps, red regions correspond to electron-rich, nucleophilic domains, blue regions denote electron-deficient, electrophilic sites, and green/yellow regions indicate areas of near-neutral potential. Compound **3** exhibits moderately negative potential localized around the acetyl carbonyl oxygen and triazole nitrogens, whereas the dichlorophenyl ring remains largely neutral, yielding an overall balanced electronic landscape. In contrast, chalcone **4b** displays pronounced regions of negative potential concentrated on the nitro substituent and α,β-unsaturated carbonyl, indicative of substantial intramolecular polarization and an enhanced electron-withdrawing character. Hydrazone **5** demonstrates the most intense electron-rich zones among the examined structures, particularly around the thioamide sulfur and hydrazone nitrogen, producing a distinctly polarized and highly nucleophilic segment within the molecule. Conversely, pyrazole **11** shows a more broadly distributed negative potential across the heterocyclic nitrogens and nitro group, generating a multi-centered electron-density pattern typical of heteroatom-enriched systems. Collectively, the MEP surfaces delineate clear electronic distinctions among the chemotypes, underscoring the role of functional-group architecture in shaping the overall electrostatic environment. For reference, novobiocin, the co-crystallized ligand, contains multiple polar and hydrogen-bonding groups that enable extensive electrostatic interactions within the enzyme active site. Gentamicin exhibits a highly cationic MEP surface characterized by extensive positive potential over its protonated amino groups, with limited negative regions around hydroxyl oxygens. This pronounced polarity reflects its strong electrostatic interactions with negatively charged biological targets. In comparison, the synthesized triazole derivatives exhibit more localized charge distributions, reflecting differences in interaction patterns while maintaining a balance between binding and membrane permeability.

#### Physicochemical and ADME analysis

To further rationalize the observed biological activity, selected physicochemical and ADME descriptors were calculated using the SwissADME web tool for representative compounds (**3**, **4b**, **5**, and **11**), as illustrated in Table 4. The calculated parameters included lipophilicity (consensus logP), topological polar surface area (TPSA), gastrointestinal (GI) absorption, P-glycoprotein (P-gp) substrate prediction, and aqueous solubility.

**Table 4:**
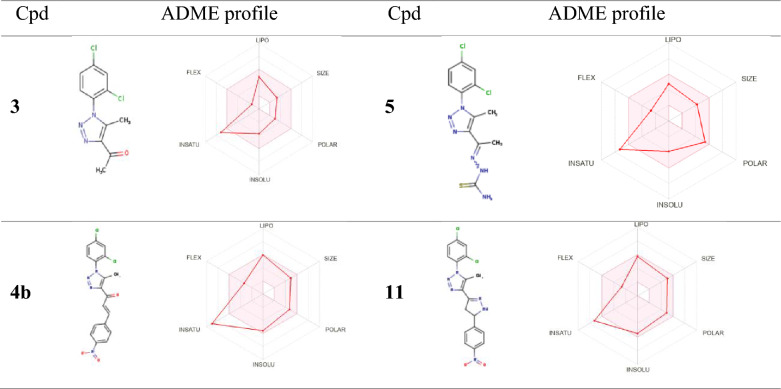
SwissADME bioavailability radar plots of compounds **3**, **4b**, **5**, and **11**.

As summarized in Table 5, compound **3** exhibited a favorable balance between lipophilicity (logP = 2.84) and polarity (TPSA = 47.78 Å^2^), which is consistent with efficient membrane permeability^28^. In contrast, compounds **4b**, **5**, and **11** displayed significantly higher polarity (TPSA = 93.60, 113.21, and 100.92 Å^2^, respectively), which may limit their ability to penetrate bacterial cell membranes despite moderate lipophilicity values.

**Table 5:** Physicochemical and ADME properties of selected compounds (**3**, **4b**, **5**, and **11**) calculated using SwissADME.

Compound	Consensus logP	TPSA (Å^2^)	GI absorption	P-gp substrate
**3**	2.84	47.78	High	No
**4b**	3.61	93.60	High	No
**5**	2.63	113.21	High	No
**11**	3.18	100.92	High	No

All tested compounds showed high predicted GI absorption and were not identified as P-gp substrates, suggesting that efflux is unlikely to be a major limiting factor. However, differences in polarity appear to play a critical role in modulating whole-cell antimicrobial activity. In particular, compounds with excessively high TPSA values (> 90 Å^2^) may exhibit reduced membrane permeability, which could explain their lower biological activity compared to compound **3**.

Overall, these results support the hypothesis that an optimal balance between lipophilicity and polarity is essential for achieving effective antimicrobial activity, complementing the insights obtained from DFT and molecular docking analyses.

## Materials and methods

### Chemistry

Melting points were determined using a Gallenkamp melting point apparatus and are reported without correction. FT-IR spectra were recorded on a Pye-Unicam SP-3–300 infrared spectrophotometer and are presented as absorption bands in cm^–1^. ^1^H NMR spectra were acquired at 400 MHz on a Varian Gemini spectrometer, using tetramethylsilane (TMS) as an internal reference and DMSO-d₆ as the solvent. Chemical shifts (δ) are reported in ppm relative to the solvent signal, and coupling constants (J) are given in Hz.

Elemental (CHN) analyses were carried out using a CHN analyzer, and the obtained values were within ± 0.4% of the calculated compositions. Reaction progress was monitored by thin-layer chromatography (TLC) using Merck silica gel 60 F_254_ plates, with visualization under UV light. Compound **3** was previously reported in the literature^29^.

#### Preparation of 1-azido-2,4-dichlorobenzene (2)

2,4-dichloroaniline **1** (1.60 g, 10 mmol) was dissolved in 2 N HCl, stirred at 0 °C till complete solubility. Sodium nitrite (0.65 g, 10 mmol) was dissolved in cold water and added to the reaction mixture. After complete addition, sodium azide (1.3 g, 200 mmol) dissolved in the least amount of water was added to the reaction mixture. The reaction mixture was stirred at r.t. for ≈1 h. The resulting residue was collected by filtration to give the target compound **2** in 80% yield that was used directly in the next step.

#### Synthesis of 1-(1-(2,4-dichlorophenyl)-5-methyl-1H-1,2,3-triazol-4-yl)ethan-1-one (3)

The 1-azido-2,4-dichlorobenzene **2** (1.88 g, 10 mmol), acetylacetone (1.1 ml, 10 mmol), potassium carbonate (1.39g, 10 mmol), and absolute ethanol (100 ml) were taken in a round bottomed flask equipped with stirrer. The reaction mixture was agitated at 75 °C for 4 h. After the completion of the reaction, the solvent was removed under vacuum. The residual mass was quenched in an ice–water mixture and neutralized with 10% HCl solution. The solid residue was isolated to afford compound **3**.

#### 1-(1-(2,4-dichlorophenyl)-5-methyl-1H-1,2,3-triazol-4-yl)ethan-1-one (3)

Yield 76%;browne cryslal; mp 90–92 °C (EtOH); FT-IR (KBr, *ν* /cm^-1^): 3081(CH_arom._), 2924 (CH_aliph_), 1686 (C = O); ^1^H NMR (DMSO, 400 MHz) δ 2.37 (s, 3H, CH_3_-acetyl), 2.65 (s, 3H, CH_3_), 7.74 (d, 1H, H-Ar, *J* = 8), 7.78 (d, 1H, H-Ar, *J* = 8), 8.06 (s, 1H, H-Ar); Anal. calcd. for C_11_H_9_Cl_2_N_3_O (270.11): C, 48.91; H, 3.36; N, 15.56. Found: C, 48.78; H, 3.31; N, 15.50.

#### General procedure for the synthesis compounds (4a-d)

A mixture of compound **3** (2.70 g, 10 mmol) and aromatic aldehydes namely, *p*- chlorobenzaldehyde, *p*- nitrobenzaldehyde, *p*- methoxybenzaldehde and furfuraldehyde 10 mmol) in ethanol (40 mL) was added slowly to an aqueous solution of sodium hydroxide (26 mmol) in water (15 mL). The reaction mixture was stirred at 20–25 °C for 4 h. The mixture was then filtered and the solid was washed with cold water to give compound **4a-d**.

#### 3-(4-chlorophenyl)-1-(1-(2,4-dichlorophenyl)-5-methyl-1H-1,2,3-triazol-4-yl)prop-2-en-1-one (4a)

Yield 77%; pale brown crystal; m.p. 230–323 °C (EtOH/ Dioxane); FT-IR (KBr, *ν* /cm^-1^): 3084(CH_arom._), 2927 (CH_aliph_),1660 (C = O); ^1^H -NMR (DMSO, 400 MHz) δ 2.46 (s, 3H, CH_3_), 7.54 (d, 2H, H-Ar, *J* = 8), 7.77 (d, 1H, H-Ar, *J* = 8), 7.83 (d, 1H, *J* H–H = 16 Hz, -CH = CH), 7.89–7.91 (m, 3H, H-Ar), 8.01 (s, 1H, H-Ar), 8.05 (d, 1H, *J* H–H = 16 Hz, -CH = CH); Anal. calcd. for C_18_H_12_Cl_3_N_3_O (392.66): C, 55.06; H, 3.08; N, 10.70. Found: C, 54.89; H, 3.00; N, 10.62.

#### 1-(1-(2,4-dichlorophenyl)-5-methyl-1H-1,2,3-triazol-4-yl)-3-(4-nitrophenyl) prop-2-en-1-one (4b)

Yield 71%; brown crystal; m.p. 267–269 °C (EtOH); FT-IR (KBr, *ν* /cm^-1^): 3082 (CH_arom._), 2851 (CH_aliph_), 1664 (C = O); ^1^H -NMR (DMSO, 400 MHz) δ 2.48 (s, 3H, CH_3_), 7.77(d, 1H, H- Ar, *J* = 8), 7.84 (d, 1H, H-Ar, *J* = 8), 7.95 (d, 1H, *J* H–H = 16 Hz, -CH = CH), 8.10 (s, 1H, H-Ar), 8.14 (d, 2H, H-Ar, *J* = 8), 8.16 (d, 1H, *J* H–H = 16 Hz, -CH = CH), 8.30 (d, 2H, H-Ar, *J* = 8); Anal. calcd. for C_18_H_12_Cl_2_N_4_O_3_(403.22): C, 53.62; H, 3.00; N, 13.90. Found: C, 53.47; H, 2.95; N, 13.81.

#### 1-(1-(2,4-dichlorophenyl)-5-methyl-1H-1,2,3-triazol-4-yl)-3-(4-methoxyphenyl)prop-2-en-1-one(4c)

Yield 48%; yellow crystal; m.p. 148–150 °C(EtOH); FT-IR (KBr, *ν* /cm^-1^): 3071 (CH_arom_), 2860 (CH_aliph_), 1658 (C = O); ^1^H -NMR (DMSO, 400 MHz) δ 2.45 (s, 3H, CH_3_), 3.83 (s, 3H, OCH_3_), 7.03(d, 2H, H- Ar, *J* = 8), 7.75 (d, 1H, H-Ar, *J* = 8), 7.78–7.83 (m, 3H, H-Ar), 7.87 (d, 2H, H-Ar, *J* = 8 Hz), 8.05 (s, 1H, H-Ar); Anal. calcd. for C_19_H_15_Cl_2_N_3_O_2_(388.25): C, 58.78; H, 3.89; N, 10.82. Found: C, 58.66; H, 3.85; N, 10.78.

#### 1-(1-(2,4-dichlorophenyl)-5-methyl-1H-1,2,3-triazol-4-yl)-3-(furan-2-yl)prop-2-en-1-one (4d)

Yield 51%; brown crystal; m.p. 159–161 °C(EtOH/ Dioxane); FT-IR (KBr, *ν* /cm^-1^): 3055 (CH_arom._), 2963 (CH_aliph_), 1656 (C = O); ^1^H -NMR (DMSO, 400 MHz) δ 2.45 (s, 3H, CH_3_), 7.20–7.22 (dd, 1H, H_Furan_), 7.68 (d, 1H, H_Furan_), 7.69 (d, 1H, *J* H–H = 16 Hz, -CH = CH), 7.74 (d, 1H, H_Furan_), 7.80 (d, 1H, H-Ar, *J* = 8), 7.82 (d, 1H, H-Ar, *J* = 8), 8.03 (d, 1H, *J* H–H = 16 Hz, -CH = CH), 8.05 (s, 1H, H-Ar); Anal. calcd. for C_16_H_11_Cl_2_N_3_O_2_ (348.18): C, 55.19; H, 3.18; Cl, 20.36; N, 12.07. Found: C, 55.00; H, 3.09; N, 11.99.

#### General procedure for the synthesis compounds (5–8)

Amixtuer of compound **3** (2.70 g, 10 mmol) and hydrazide derivatives namely, thiosemicarbazied, semicarbaziedhydrochloride, ethylcarbazate and 2,4-dinitrophenylhydrazine (10 mmmol) in ethanol (20 mL) with catalytic amount of acetic acid (5mL) in **5**, **7**, and **8** or sodium acetate in **6** was refluxed. The progress of the reaction was checked by TLC. After cooling, the resulting precipitated solid was collected by filtration, washed with water, dried, and then purified by recrystallization from ethanol to give pure products (**5**–**8**).

#### Synthesis of (E)-2-(1-(1-(2,4-dichlorophenyl)-5-methyl-1H-1,2,3-triazol-4-yl)ethylidene) hydrazine-1-carbothioamide (5)

Yield 68%; off white crystal; m.p. 238–240 °C (EtOH/ Dioxane); FT-IR (KBr, *ν* /cm^-1^): 3443, 3284, 3211 (NH, NH_2_), 3053 (CH_arom._), 2987 (CH_aliph_); ^1^H-NMR (400 MHz, DMSO-*d*_6_) *δ* (ppm): 2.36 (s, 3H, CH_3_), 2.49 (s, 3H, CH_3_), 7.38 (s, 1H, = NH, exchangeable D_2_O), 7.71 (d, 1H, H-Ar, *J* = 8), 7.73 (d, 1H, H-Ar, *J* = 8), 8.01 (s, 1H, Ar–H), 8.35 (s, 1H, SH, exchangeable D_2_O), 10.29 (s,1H, NH, exchangeable D_2_O); ^13^C-NMR (100 MHz, DMSO-*d*_6_) *δ* (ppm): 179.40, 145.10, 142.05, 140.03, 136.90, 134.95, 132.24, 131.36, 130.60, 129.34, 14.99, 10.62; Anal. Calcd. for C_12_H_12_Cl_2_N_6_S (343.23): C, 41.99; H, 3.52; N, 24.49. Found: C, 32.00; H, 3.48; N, 24.41.

#### Synthesis of (E)-2-(1-(1-(2,4-dichlorophenyl)-5-methyl-1H-1,2,3-triazol-4-yl)ethylidene) hydrazine-1-carboxamide (6)

Yield 77%; pale brown crystal; m.p. 254–256 °C(EtOH/Dioxane); FT-IR (KBr, *ν* /cm^-1^): 3293, 3186, 3145 (NH, NH_2_), 3081 (CH_arom._), 1673 (C = O); ^1^H-NMR (400 MHz, DMSO-*d*_6_) *δ* (ppm): 2.34 (s, 3H, CH_3_), 2.38 (s, 3H, CH_3_), 6.35 (s, 2H, NH_2_, exchangeable D_2_O), 7.70 (d, 1H, H-Ar, *J* = 8), 7.73 (d, 1H, H-Ar, *J* = 8), 8.01 (s, 1H, Ar–H), 9.58 (s, 1H, NH, exchangeable D_2_O); Anal. Calcd. for C_12_H_12_Cl_2_N_6_O (327.17): C, 44.05; H, 3.70; N, 25.69. Found: C, 34.05; H, 3.65; N, 25.60.

#### Synthesis of ethyl (E)-2-(1-(1-(2,4-dichlorophenyl)-5-methyl-1H-1,2,3-triazol-4-yl)ethylidene)hydrazine-1-carboxylate (7)

Yield 64%; white crystal; m.p. 149–151 °C (pet-80–100); FT-IR (KBr, *ν* /cm^-1^): 3288, (NH), 3082 (CH_arom._), 2921(CH_aliph_) 1708 (C = O); ^1^H-NMR (400 MHz, DMSO-*d*_6_) *δ* (ppm): 1.23 (t, 3H, -CH_2_CH_3_, *J* = 8), 2.37 (s, 3H, CH_3_), 2.38 (s, 3H, CH_3_), 4.14–4.19 (q, 2H, -CH_2_CH_3_), 7.74–7.78 (m, 2H, H-Ar), 8.05 (s, 1H, H-Ar), 10.24 (s, 1H, NH, exchangeable D_2_O); ^13^C-NMR (100 MHz, DMSO-*d*_6_) *δ* (ppm): 154.58, 144.79, 142.42, 136.82, 134.43, 132.49, 132.27, 131.41, 130.62, 129.34, 61.08, 14.99, 14.28, 10.02; Anal. Calcd. for C_14_H_15_Cl_2_N_5_O_2_ (356.21): C, 47.11; H, 4.19; N, 19.60. Found: C, 47.21; H, 4.24; N, 19.66.

#### Synthesis of (E)-1-(2,4-dichlorophenyl)-4-(1-(2-(2,4-dinitrophenyl)hydrazineylidene)ethyl)-5-methyl-1H-1,2,3-triazole (8)

Yield 79%; orange crystal; m.p. 264–266 °C (Dioxane); FT-IR (KBr, *ν* /cm^-1^): 3238 (NH), 3093 (CH_arom._), 2920(CH_aliph_); ^1^H-NMR (400 MHz, DMSO-*d*_6_) *δ* (ppm): 2.45 (s, 3H, CH_3_), 2.58 (s, 3H, CH_3_), 7.44 (d, 1H, H-Ar, *J* = 8),7.79 (d, 1H, H-Ar, *J* = 8), 7.83 (d, 1H, H-Ar, *J* = 8), 8.12 (s, 1H, Ar–H), 8.19 (d, 1H, H-Ar, *J* = 8), 8.93 (s, 1H, Ar–H), 14.34 (s, 1H, NH, exchangeable D_2_O); Anal. Calcd. for C_17_H_13_Cl_2_N_7_O_4_(450.24): C, 45.35; H, 2.91; N, 21.78. Found: C, 45.22; H, 2.87; N, 21.70.

#### General procedure for preparation of 9a, 10 and 11

To a solution of chalcone **4a** (3.92 g, 10 mmol) or **4b** (4.02 g, 10 mmol) in acetic acid (10 mL) or formic acid (10 mL) or in ethanol (15 mL), hydrazine hydrate (20 mmol, 1 mL) was added. The reaction mixture was refluxed for 6 h (monitored by TLC)., The reaction mixture was cooled and poured onto crushed ice. The obtained precipitation was filtered, washed with H_2_O, dried, and recrystallized from appropriate solvent to afford compounds **9a, 10,** and **11**.

#### Synthesis of 1-(5-(4-chlorophenyl)-3-(1-(2,4-dichlorophenyl)-5-methyl-1H-1,2,3-triazol-4-yl)-4,5-dihydro-1H-pyrazol-1-yl)ethan-1-one (9a)

Yield 75%; brown crystal; m.p. 169–1171 °C (EtOH/ Dioxane); FT-IR (KBr, *ν* /cm^-1^): 3082 (CH_arom._), 2851 (CH_aliph_), 1657 (C = O). ^1^H-NMR (400 MHz, DMSO-*d*_6_) *δ* (ppm): 2.29 (s, 3H, CH_3_), 2.46 (s, 3H, CH_3_), 3.19–3.25 (dd, 1H, CH_2_-pyrazole), 3.99- 4.06 (dd, 1H, CH_2_-pyrazole), 5.54–5.58 (dd, 1H, CH-pyrazole), 7.25 (d, 2H, H-Ar, *J* = 8), 7.39 (d, 2H, H-Ar, *J* = 8), 7.74 (d, 1H, H-Ar, *J* = 8), 7.78 (d, 1H, H-Ar, *J* = 8), 8.05 (s, 1H, Ar–H). Anal. Calcd. for C_20_H_16_Cl_3_N_5_O (448.73): C, 53.53; H, 3.59; N, 15.61. Found: C, 53.43; H, 3.55; N, 15.55.

#### Synthesis of 5-(4-chlorophenyl)-3-(1-(2,4-dichlorophenyl)-5-methyl-1H-1,2,3-triazol-4-yl)-1H-pyrazole-1-carbaldehyde (10)

Yield 64%; white crystal; m.p. 98–100 °C (EtOH); FT-IR (KBr, *ν* /cm^-1^): 3053 (CH_arom._), 2932 (CH_aliph_), 1676 (C = O). ^1^H-NMR (400 MHz, DMSO-*d*_6_) *δ* (ppm): 2.41 (s, 3H, CH_3_), 3.25–3.30 (dd, 1H, CH_2_-pyrazole), 4.04- 4.12 (dd, 1H, CH_2_-pyrazole), 5.53–5.57 (dd, 1H, CH-pyrazole), 7.29–8.07 (m, 7H, H-Ar), 8.90 (s,1H, CHO); ^13^C-NMR (100 MHz, DMSO-*d*_6_) *δ* (ppm): 160.27, 150.96, 140.46, 137.12, 136.89, 136.11, 132.60, 132.18, 132.03, 131.39, 130.73, 129.45, 129.22 (2C), 128.26 (2C), 57.39, 43.82, 9.78; Anal. Calcd. for C_19_H_12_Cl_3_N_5_O (432.69): C, 52.74; H, 2.80; N, 16.19. Found: C, 52.63; H, 2.75; N, 16.12.

#### Synthesis of 1-(2,4-dichlorophenyl)-5-methyl-4-(5-(4-nitrophenyl)-1H-pyrazol-3-yl)-1H-1,2,3-triazole (11)

Yield 60%; pale yellow powder; m.p. 92–94 °C (EtOH); FT-IR (KBr, *ν* /cm^-1^): 3342 (NH), 3081 (CH_arom._), 29240 (CH_aliph_); ^1^H-NMR (400 MHz, DMSO-*d*_6_) *δ* (ppm): 2.36 (s, 3H, CH_3_), 2.99–3.04 (dd, 1H, CH_2_-pyrazole), 3.69- 3.76 (dd, 1H, CH_2_-pyrazole), 4.99–5.05 (m, 1H, CH-pyrazole), 7.69–7.75 (m, 5H, H-Ar), 7.76 (s. 1H, NH, exchangeable D2O)7.78–8.25 (m, 2H, H-Ar); Anal. Calcd. for C_18_H_12_Cl_2_N_6_O_2_(415.23): C, 52.07; H, 2.91; N, 20.24. Found: C, 51.89; H, 2.87; N, 20.18.

#### Synthesis of 1-(3-(1-(2,4-dichlorophenyl)-5-methyl-1H-1,2,3-triazol-4-yl)-5-(4-nitrophenyl)-1H-pyrazol-1-yl)ethan-1-one (9b)

To a solution of chalcone **4b** (4.02 g, 10 mmol) in acetic acid (10 ml) hydrazine hydrate (20 mmol, 1 mL) was added. The reaction mixture was refluxed for 6 h (monitored by TLC). The reaction mixture was cooled and poured onto crushed ice. The obtained precipitation was filtered, washed with H_2_O, dried, and recrystallized from appropriate solvent to afford compounds **9b.**

Yield 65%; off white crystal; m.p. 110–113 °C (EtOH/ Dioxane); FT-IR (KBr, *ν* /cm^-1^): 3080 (CH_arom._), 2930 (CH_aliph_), 1666 (C = O). ^1^H-NMR (400 MHz, DMSO-*d*_6_) *δ* (ppm): 2.31 (s, 3H, CH_3_), 2.46 (s, 3H, CH_3_), 3.23–3.33 (dd, 1H, CH_2_-pyrazole), 4.05- 4.12 (dd, 1H, CH_2_-pyrazole), 5.66–5.71 (d, 1H, CH-pyrazole), 7.51–8.23 (m, 7H, H-Ar); ^13^C-NMR (100 MHz, DMSO-*d*_6_) *δ* (ppm): 168.24, 149.90, 149.05, 147.21, 137.11, 137.01, 136.02, 132.19, 132.06, 131.39, 130.73, 129.45, 127.50 (2C), 124.46 (2C), 58.54, 43.45, 22.03, 9.76; Anal. Calcd. for C_20_H_14_Cl_2_N_6_O_3_ (457.27): C, 52.53; H, 3.09; N, 18.38. Found: C, 52.45; H, 3.03; N, 18.30.

#### Synthesis of (E)-2-((E)-3-(4-chlorophenyl)-1-(1-(2,4-dichlorophenyl)-5-methyl-1H-1,2,3-triazol-4-yl)allylidene)hydrazine-1-carbothioamide (12)

To the chalcone 4b (4.02 g, 10 mmol) and catalytic amount of acetic acid (5 mL) in ethanol (40 mL), thiosemicarbazide (0.91 g, 10 mmol) was added. The mixture was refluxed for 6 h (monitored by TLC)., then left to cool, the solid product was filtered off, washed with ethanol, dried, recrystallized from proper solvent to give compound **12**.

Yield 73%; pale yellow crystal; m.p. 128–130 °C (EtOH); FT-IR (KBr, *ν* /cm^-1^): 3428, 3259, 3152 (NH, NH_2_), 2961 (CH_arom._), 1663 (C = O). ^1^H-NMR (400 MHz, DMSO-*d*_6_) *δ* (ppm): 2.16 (s, 3H, CH_3*,*_* E*-Isomer), 2.26 (s, 3H, CH_3,_ *z*-isomer), 6.97 (d, 1H, *J* H–H = 16.4 Hz, -CH = CH), 7.18 (d, 1H, *J* H–H = 16.4 Hz, -CH = CH), 7.27–8.64 (m, 16H, H-Ar, *Z* & *E* mixture), 10.71 (s, 1H, NH, *E*-isomer), 11.42 (s, 1H, NH, *Z*-isomer); Anal. calcd. for C_19_H_15_Cl_3_N_6_S (465.78): C, 48.99; H, 3.25; N, 18.04. Found: C, 48.87; H, 3.21; N, 17.98.

### Molecular docking

Molecular docking studies were performed to investigate the binding interactions of the synthesized compounds with *Staphylococcus aureus* topoisomerase IV (PDB ID: 4URN). The chemical structures of all ligands were initially sketched using ChemDraw, converted into three-dimensional geometries, and subsequently energy-minimized using the MMFF94 force field prior to docking using Chem3D.

The crystal structure of the target enzyme was retrieved from the Protein Data Bank and prepared for docking by removing co-crystallized ligands and water molecules, followed by the addition of polar hydrogen atoms and assignment of appropriate partial charges^30,31^. Docking simulations were carried out using AutoDock Vina, employing a grid box centered on the active site corresponding to the binding pocket of the co-crystallized ligand^32^. Default Vina parameters were used, and multiple binding poses were generated for each ligand.

To validate the docking protocol, the co-crystallized ligand (novobiocin) was re-docked into the active site, and the root-mean-square deviation (RMSD) between the experimental and predicted binding poses was found to be less than 2.0 Å, indicating good agreement and reliability of the docking procedure. The same protocol was subsequently applied to all synthesized compounds.

The resulting docking conformations were ranked based on their predicted binding affinities (kcal/mol), and the most favorable binding pose for each compound was selected for further analysis^33^. Protein–ligand interactions, including hydrogen bonding, π–π stacking, and hydrophobic contacts, were analyzed and visualized using PyMOL.

This docking protocol enabled a comparative assessment of binding modes and affinities across the compound series and provided molecular-level insights correlating with the observed antimicrobial activity.

### Antimicrobial

The antimicrobial activity of all synthesized compounds against Gram-positive bacteria *Escherichia coli* (ATCC 25922), and *Helicobacter pylori (*ATCC 43526*).* Gram-negative bacteria *Bacillus cereus* (ATCC 6629), and *Staphylococcus aureus* (ATCC 6538), and Pathogenic fungi *Candida albicans* (ATCC 10231) were evaluated using inhibition zone diameter measurements^34^, as described in the Supporting Information. All antimicrobial assays were performed in triplicate (n = 3) to ensure reproducibility. The inhibition zone diameters were measured directly from agar plates, and the observed variation between independent measurements was minimal (typically within ±1 mm). Therefore, representative values are reported.

### DFT calculations

Geometry optimizations were performed using density functional theory (DFT) with the B3LYP hybrid functional in conjunction with the 6-311+G(d,p) basis set, as implemented in the Gaussian 09 program package^35,36^. This level of theory is widely employed for reliable prediction of molecular geometries and electronic properties of heterocyclic systems and drug-like molecules^37^. Geometry optimizations were carried out using standard convergence criteria without symmetry constraints. Frequency calculations were performed at the same level of theory to confirm that the optimized structures correspond to true minima. All calculations were performed in the gas phase.

## Conclusion

A series of 2,4-dichlorophenyl–1,2,3-triazole derivatives incorporating diverse pharmacophoric moieties was successfully synthesized and evaluated for antimicrobial activity. Among them, compound **3** demonstrated the most consistent broad-spectrum activity, highlighting the importance of the triazole–acetyl scaffold. Structure–activity relationship and ADME analyses revealed that a balance between lipophilicity and polarity is critical for effective antimicrobial performance. Molecular docking and DFT studies provided complementary insights into binding interactions and electronic properties, supporting the experimental findings while emphasizing their qualitative role. Overall, the 2,4-dichlorophenyl–1,2,3-triazole framework represents a promising starting point for further optimization of antimicrobial agents.

## Supplementary Information

Supplementary Information.

## Data Availability

The datasets generated and/or analysed during the current study are included in this published article and its Supplementary Information files.
