# Supplementary material for: Rational design, synthesis, and antimicrobial evaluation of novel 1,2,3-triazole hybrids: structure–activity relationships, molecular docking, and DFT studies
Source: Sci Rep. 2026 Aug 11;16:24903. doi: 10.1038/s41598-026-62695-w (PMC13458703; doi:10.1038/s41598-026-62695-w)
Supplement: Supplementary file 1 [file 41598_2026_62695_MOESM1_ESM.docx]

**Rational Design, Synthesis, and Antimicrobial Evaluation of Novel 1,2,3-Triazole Hybrids: Structure–Activity Relationships, Molecular Docking, and DFT Studies**

Mohammed N. Sallam^1^, A. M. A. Hassan^2^, Abdullah Yahya Abdullah Alzahrani ^3^, Hossam M. El-Masry^4^, Abeer M. El-Naggar^2^, Eslam M. Abbass^2*^

^1^ Egypt Otsuka Pharmaceutical, Tenth of Ramadan, Egypt.

^2^ Department of Chemistry, Faculty of Science, Ain Shams University, Abbassia, 11566, Cairo, Egypt.

^3^Department of Chemistry, Faculty of Science, King Khalid University, Abha 61413, Saudi Arabia

^4^Chemistry of Natural and Microbial Products department, Pharmaceutical and Drug Industries Research Institute.

* Correspondence:

**Eslam M. Abbass**

**Email:** [eslammorad@sci.asu.edu.eg](mailto:eslammorad@sci.asu.edu.eg)

**ORCID:** [0000-0002-3245-1653](https://orcid.org/0000-0002-3245-1653)

**Figure S1.** IR spectrum of compound **3**

**Figure S2.** ^1^H NMR (400 MHz, DMSO-*d_6_*) spectrum of compound **3**

**Figure S3.** IR spectrum of compound **4a**

**Figure S4.** ^1^H NMR (400 MHz, DMSO-*d_6_*) spectrum of compound **4a**

**Figure S5.** IR spectrum of compound **4b**

**Figure S6.** ^1^H NMR (400 MHz, DMSO-*d_6_*) spectrum of compound **4b**

**Figure S7.** IR spectrum of compound **4c**

**Figure S8.** ^1^H NMR (400 MHz, DMSO-*d_6_*) spectrum of compound **4c**

**Figure S9.** IR spectrum of compound **4d**

**Figure S10.** ^1^H NMR (400 MHz, DMSO-*d_6_*) spectrum of compound **4d**

**Figure S11.** IR spectrum of compound **5**

**Figure S12.** ^1^H NMR (400 MHz, DMSO-*d_6_*) spectrum of compound **5**

**Figure S13.** ^13^C NMR (100 MHz, DMSO-*d_6_*) spectrum of compound **5**

**Figure S14.** IR spectrum of compound **6**

**Figure S15.** ^1^H NMR (400 MHz, DMSO-*d_6_*) spectrum of compound **6**

**Figure S16.** IR spectrum of compound **7**

**Figure S17.** ^1^H NMR (400 MHz, DMSO-*d_6_*) spectrum of compound **7**

**Figure S18.** ^13^C NMR (100 MHz, DMSO-*d_6_*) spectrum of compound **7**

**Figure S19.** IR spectrum of compound **8**

**Figure S20.** ^1^H NMR (400 MHz, DMSO-*d_6_*) spectrum of compound **8**

**Figure S21.** IR spectrum of compound **9a**

**Figure S22.** ^1^H NMR (400 MHz, DMSO-*d_6_*) spectrum of compound **9a**

**Figure S23.** IR spectrum of compound **9b**

**Figure S24.** ^1^H NMR (400 MHz, DMSO-*d_6_*) spectrum of compound **9b**

**Figure S25.** ^13^C NMR (100 MHz, DMSO-*d_6_*) spectrum of compound **9b**

**Figure S26.** IR spectrum of compound **10**

**Figure S27.** ^1^H NMR (400 MHz, DMSO-*d_6_*) spectrum of compound **10**

**Figure S28.** ^13^C NMR (100 MHz, DMSO-*d_6_*) spectrum of compound **10**

**Figure S29.** IR spectrum of compound **11**

**Figure S30.** ^1^H NMR (400 MHz, DMSO-*d_6_*) spectrum of compound **11**

**Figure S31.** IR spectrum of compound **12**

**Figure S32.** ^1^H NMR (400 MHz, DMSO-*d_6_*) spectrum of compound **12**

**Table (S1):** Inhibition zone diameter (millimeter) of the samples applying the well diffusion method: -

| **Bacteria**  **Samples** | | **Test bacteria** | | | | |
| --- | --- | --- | --- | --- | --- | --- |
|  |  | ***Escherichia***  ***coli*** | ***Helicobacter***  ***pylori*** | ***Bacillus***  ***cereus*** | ***Staphylococcus***  ***aureus*** | ***Candida***  ***albicans*** |
| **1-** | **10** | **NIL*** | **NIL** | **NIL** | **12.0** | **11.0** |
| **2-** | **7** | **15.0** | **NIL** | **NIL** | **NIL** | **13.0** |
| **3-** | **5** | **12.0** | **10.0** | **10.0** | **14.0** | **NIL** |
| **4-** | **6** | **11.0** | **NIL** | **NIL** | **NIL** | **NIL** |
| **5-** | **9b** | **10.0** | **NIL** | **NIL** | **NIL** | **NIL** |
| **6-** | **4a** | **10.0** | **NIL** | **NIL** | **NIL** | **10.0** |
| **7-** | **4c** | **12.0** | **NIL** | **NIL** | **NIL** | **9.0** |
| **8-** | **4d** | **12.0** | **NIL** | **NIL** | **NIL** | **10.0** |
| **9-** | **4b** | **11.0** | **NIL** | **NIL** | **11.0** | **10.0** |
| **11-** | **3** | **14.0** | **13.0** | **15.0.** | **13.0** | **15.0** |
| **12-** | **11** | **13.0** | **11.0** | **10.0** | **13.0** | **NIL** |
| **13-** | **12** | **12.0** | **12.0** | **NIL** | **NIL** | **NIL** |
| **14-** | **9a** | **11.0** | **12.0** | **NIL** | **11.0** | **NIL** |
| **15-** | **8** | **NIL** | **NIL** | **NIL** | **NIL** | **NIL** |
| ****** | **CN**** | **13.0** | **20.0** | **19.0** | **18.0** | **17.0** |

**Figure S33:** Inhibition zone diameter (millimeter) of the samples applying well diffusion method: -

**Tested organisms:**

1- Gram-Negative Bacteria:

*Escherichia coli* (ATCC 25922).

*Helicobacter pylori (*ATCC 43526*).*

2- Gram-Positive Bacteria:

*Bacillus cereus* (ATCC 6629).

*Staphylococcus aureus* (ATCC 6538).

3- Pathogenic Fungi:

*Candida albicans* (ATCC 10231).

**Methods: -**

The antimicrobial activity of the synthesized compounds was evaluated against Gram-positive bacteria Bacillus cereus and Staphylococcus aureus, Gram-negative bacteria Escherichia coli and Helicobacter pylori, and the pathogenic yeast Candida albicans using the agar well diffusion method according to El-Masry et al. (2023). Fresh overnight cultures were prepared in nutrient broth and incubated at 37 °C (Abdel Raoof et al., 2024). The inoculum density was adjusted to approximately 0.5 McFarland standard (1.5 × 10⁸ CFU/mL) (McFarland, 1907). A volume of 25.0 µL of each microbial suspension was inoculated onto sterile nutrient agar plates (50.0 mL per plate). Wells of 0.6 cm diameter were prepared using a sterile cork borer, and each well was filled with 75.0 µL of the test compound solution (50.0 mg/mL). Gentamicin was used as the positive control for antibacterial assays at a concentration of 100 µg/mL, prepared in sterile distilled water and applied under identical conditions. The plates were kept at 4 °C for 1 h to allow diffusion, followed by incubation at 37 °C for 24 h. The inhibition zones were measured in millimeters (mm). The minimum inhibitory concentration (MIC) of the most active compound was determined using the broth microdilution method (Abdel Raoof et al., 2024). The assay was carried out in a 96-well microplate, where each well contained 100.0 µL of nutrient broth. Serial two-fold dilutions of the compound were prepared starting from 50.0 mg/mL. A volume of 100.0 µL of the microbial inoculum (0.5 McFarland standard, 1.5 × 10⁸ CFU/mL) was added to each well to obtain a final volume of 200 µL. The plates were incubated at 37 °C for 24 h. Gentamicin was evaluated in parallel as the reference antibacterial standard using the same microdilution conditions. Serial dilutions were prepared under identical conditions. After incubation, microbial growth was assessed by measuring the absorbance at 600 nm using a microplate reader (Bio-Rad Laboratories Inc., model 3350, Hercules, CA, USA). The MIC was defined as the lowest concentration showing no visible growth (absorbance comparable to the blank control).

**ADME profiles:**

**Figure S34.** SwissADME profile of compound **3**.

**Figure S35.** SwissADME profile of compound **4b**.

**Figure S36.** SwissADME profile of compound **5**.

**Figure S37.** SwissADME profile of compound **11**.

**References:**

1) Abdel Raoof, G. F., Abuaish, M. A. A., & M. El-Masry, H. (2024). Assessment of Vicia faba L. Peels: Phytochemical Characterization and Evaluation of Cytotoxic and Antimicrobial Potentials. *Chemistry* & *Biodiversity*, e202402123, <https://doi.org/10.1002/cbdv.202402123>.

‏2) El-Masry, H. Mohammed, Atwa, N., M., El-Beih, A., Agwa, M., Khafagi, I., K., Mansour, S., R. & Eldiwany, A., I. (2023): Phenazine-Producing *Pseudomonas* *aeruginosa* OQ158909: A Promising Candidate for Biological Activity and Therapeutic Applications. *Egyptian Journal of Chemistry,* 66(11), 281-303, https://doi.org/[10.21608/EJCHEM.2023.214109.8045](https://doi.org/10.21608/ejchem.2023.214109.8045).

3) Hafez, A. I., Ali, H. M., Sabry, R. M., El-Masry, H. M., & Abd El-Gawad, W. M. (2023). Generation of novel, hygienic, inhibitive, and cost-effective nanostructured Core-shell pigments. *Progress in Organic Coatings*, *175*, 107325, <https://doi.org/10.1016/j.porgcoat.2022.107325>.

4) Hamoda, D. M., Kanawy, S. H., Aboalmaged, H. H., Rashed, U. M., Almetwally, A. A., & El-Masry, H. M. (2022). Plasma Technique Application for Coating Non-Woven Fabric by (CaSiO3/CuO) Nano Particles for Biomedical Sector. *Egyptian Journal of Chemistry*, 65(8), 773-778, https://doi.org/[10.21608/ejchem.2022.128815.5705](https://doi.org/10.21608/ejchem.2022.128815.5705).

5) McFarland, J., (1907): The nephelometer: an instrument for estimating the	number of bacteria in suspensions used for calculating	the opsonic index and for vaccines. *J. Am. Med. Assoc.* 49: 1176–1178, <https://doi.org/10.1001/jama.1907.25320140022001f>.

6) Tohamy, H. A. S., & El-Masry, H. M. (2024). Fluffy-like amphiphilic graphene oxide and its effects on improving the antibacterial activity and thermal outstanding of ethyl cellulose/polyvinyl alcohol hydrogel film. *BMC chemistry*, *18*(1), 116, <https://doi.org/10.1186/s13065-024-01221-3>.
